# Optimization and establishment of laboratory rearing conditions for *Cimex lectularius* L. against variable temperature and relative humidity

**DOI:** 10.1038/s41598-024-59728-7

**Published:** 2024-04-22

**Authors:** Amartya Banerjee, Achintya Saha, Parikshit Das, Ajay Kakati, Buddhadeb Saha, Danswrang Goyary, Yangchen D. Bhutia, Sanjeev Karmakar, Sumit Kishor, Saidur Rahaman, Pronobesh Chattopadhyay

**Affiliations:** 1 Division of Pharmaceutical Technology, Defence Research Laboratory (DRL), DRDO, Tezpur, Assam 784001 India; 2 Department of Chemical Technology, University of Calcutta, Kolkata, India; 3 Department of Pharmaceutical Sciences, Faculty of Science and Engineering, Dibrugarh University, Dibrugarh, Assam 786004 India

**Keywords:** *Cimex lectularius* L., Bed bugs, Hematophagous insect, Development, Life cycle, Zoology, Diseases, Health care

## Abstract

Emerging infestations of bed bugs are affecting normal human lifestyle globally. This study has been designed to optimize the rearing conditions for *Cimex lectularius* L. (Hemiptera), to support the scientific research on them. Bed bugs have been projected onto three different temperature (20 °C, 25 °C, and 30 °C) and relative humidity (50%, 70%, and 90%) conditions to check their overall growth and survival rate. Adult mortality, weight loss, egg laying, percentage hatching, hatching initiation and completion, nymph mortality, and molting have been evaluated to optimize the best conditions. The temperature at 25 °C with 90% RH showed minimum mortality for adults (female 13.33 ± 3.33% and male 6.67 ± 3.33%) and nymphs (13.33 ± 3.33%), while maximum egg laying (40.33 ± 1.86), with highest percentage hatching (98.23 ± 0.58%). At 30 °C with 90% RH, hatching initiation and completion (5.19 ± 0.12 days and 7.23 ± 0.16 days) as well as molting initiation and completion (3.73 ± 0.12 days and 7.00 ± 0.24 days) were found to be fastest. Thus, it can be concluded that 25 °C with 90% RH is ideal for rearing of adults and 30 °C with 90% RH is appropriate for rapid growth of nymphs.

## Introduction

Climatic conditions like temperature and humidity have very profound influence on the life cycle of arthropods^1^. One such arthropod is bed bug, a nocturnal, flightless, and blood feeding ectoparasites of the Cimicidae family. Their existence was known for over thousands of years, followed humans from cave to modern civilization, and still possesses a challenging issue in the world^2,3^. Bed bug infestation was controlled post World War-II with the use of synthetic insecticides. In 1990s resurgence occurred because of insecticides resistance and spread due to global travelling^3–5^. In recent times infestation of bed bugs were reported in America, Europe, Australia, Africa, and Asia^3–6^.

There are two main species of bed bugs, *C. lectularius* (common bed bug) and *C. hemipterus* (tropical bed bug) which are mostly found in tropical, sub-tropical and temperate regions. They preferably populate more when the temperature and relative humidity is around 23–32 °C and 50–90% respectively, and can also enter to a dormant state if the temperature drops significantly^7–9^. These obligate hematophagous insects mostly stay hidden under the closest proximity to the host in daytime and become most active during the night when the host’s movements are significantly low. They feed a warm blood meal with the help of stylet in every 4–5 days for their growth and reproduction^2,9–11^.

Nowadays, it has become very necessary to control bed bugs as they are creating a very detrimental influence on human health all around the globe^12^. Their biting may cause sleeping disturbance leading to anxiety, local inflammation, itching, impetigo, and allergic reactions^13–16^. Pyrethroids and natural oils have been found to be effective for their management, but it is very challenging to control the infestations because of their cryptic behaviour and high adaptability to various physical and chemical controlling paradigms^6,17–20^.

Hence, a systematic approach to study the behaviour of bed bugs to combat their infestations is required for which culturing of this insect effectively in the laboratory environment is of utmost importance. This implies that, a proper culturing technique that results in a good qualitative and quantitative production is a major requirement for conducting research on them. Usually, bed bugs are cultured in laboratories from 20 to 30 °C with a relative humidity (RH) of 60–70%, although they have been reported to survive in a very harsh climatic condition. Researchers around the globe are finding it difficult to control their rearing in the laboratory conditions^21–23^. There are variations in the culturing techniques at different laboratories according to their needs and facilities available, but optimal conditions of temperature and relative humidity required for the maximum production of *C. lectularius* L. species of bed bugs are yet to be established. Thus, the present study has been performed to optimize the best suitable temperature and RH for the production of maximum number of bed bugs, with low mortality rate, high egg production, fastest egg hatching and quick nymphal development in the laboratory facility.

## Methodology

### Bed bugs collection and identification

Initially, bed bugs were collected from the army barracks of Solmara, Tezpur, Assam. These were mostly found in the edges and cracks of beds and mosquito nets of army personnel and taken out with the help of a fine painting brush. A total of 89 bed bugs were collected from three different locations. The adult bed bugs (10 numbers) were stored in 70% ethanol and sent for species identification in the Zoological Survey of India (ZSI), Dehradun, India. All the specimens were identified as *C. lectularius* Linnaeus, 1758 of family Cimicidae. Further, they were reared to reproduce sufficient quantities for testing.

### Laboratory rearing and maintenance of culture

**Figure 1:**
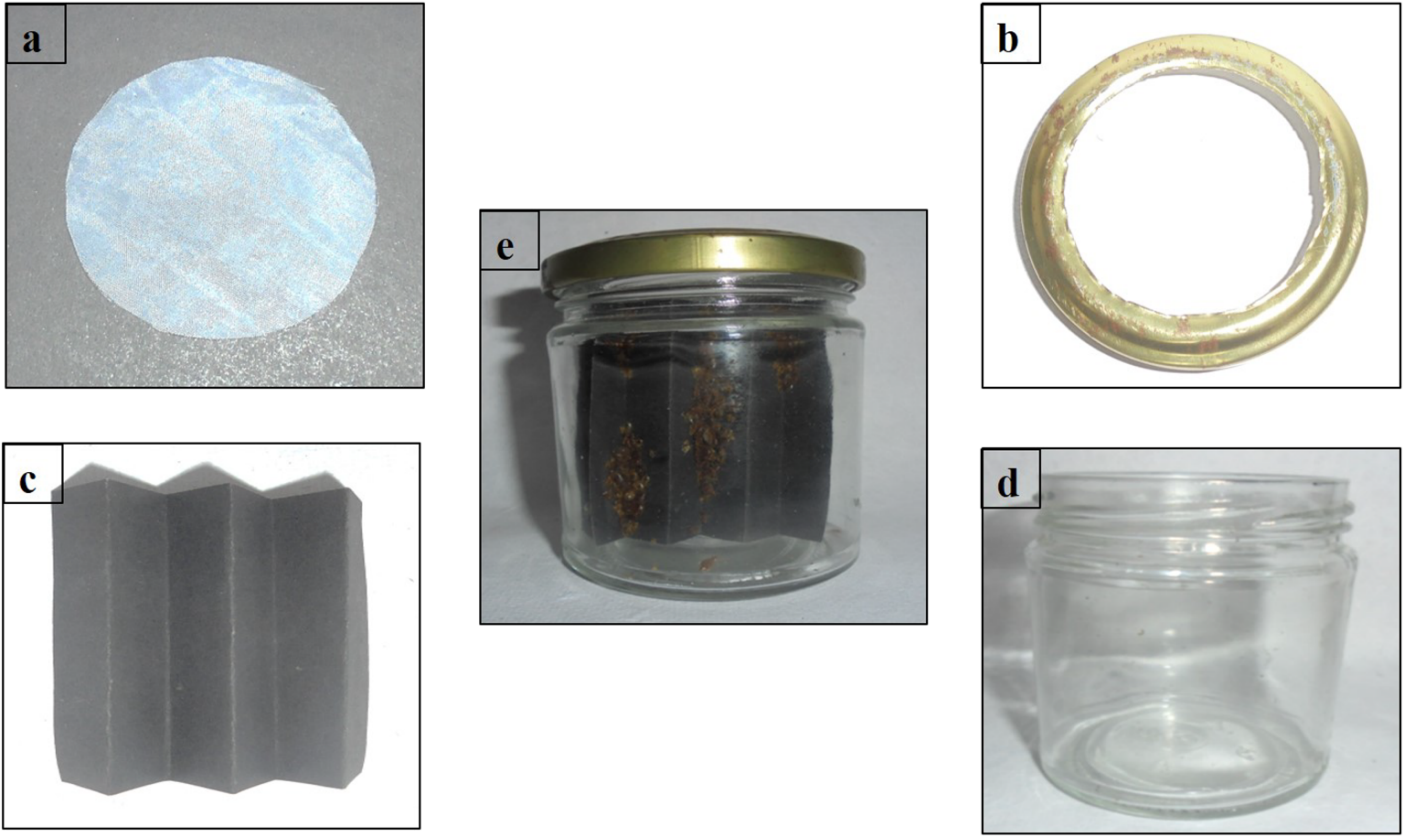
(**a**) Nylon mesh, (**b**) metal lid, (**c**) folded black chart paper for harborage, (**d**) glass jar, (**e**) bed bug culture inside the jar.

The adult bed bugs were kept at laboratory conditions (temperature 27 ± 3 °C, RH 60 ± 15%, with a 12: 12 h (L:D)) in glass jars (8 cm height and 6.5 cm inner diameter) with a tightly caped metal lid and black chart papers as harbourage (Lakeer store, Vadodara, India) (7 cm length, 6 cm width, and 5–6 folds) for hiding and ovipositing. The middle part of the lid (5 cm diameter) was removed and a nylon sheet of 120 mesh (Taher & Sons, India) was fit into the lid to promote air circulation and also to suck blood meal (in vitro rabbit blood) once weekly. The pictorial representations of different culture components are depicted in Fig. 1 below. The experimental protocol for blood withdrawal from rabbit was approved by the Institutional Animal Ethical Committee (IAEC) (approved protocol number 07, dated 25/02/2022), Defence Research Laboratory (Registration number 1227/GO/Rbi/S/08/CPCSEA), Tezpur, Assam, India. This protocol was carried out in compliance with the standards of the Animal Research: Reporting of In Vivo Experiments (ARRIVE) and CCSEA guidelines. Briefly, 5 ml blood was collected from the marginal ear vein of a healthy adult New Zealand white rabbit (male, weighing 2–2.5 kg) using a 21 G needle (DISPO VAN ®, Hindustan syringes and medical devices. LTD, India), into K3-EDTA vacutainer vials (Unitek scientific corporation, India). Then it was injected to a pouch of stretched parafilm “M” ® laboratory film (Pechiney plastic packaging, Chicago, USA), warmed at 37 °C in a water bath (Equitron, Medica Instrument Mfg. Co., India). The culture jar was kept upside down position, touching the nylon mesh to the blood-loaded stretched parafilm “M” ® pouch so that bed bugs could feed the blood^21,24^. Further, the nymphs were separated from the parent culture periodically.

### Exposure to different temperature and humidity conditions

Adult bed bugs (12–15 days old) (n = 20, Male/Female ratio 1:1) were kept in the same type of glass jars that were used for their culture. For exposure, bed bugs were kept in different temperature and RH conditions with the help of a controlled temperature and humidity cabinet (Scientech, science enterprises, Delhi, India), with a 12: 12 h (L:D). The temperature and humidity conditions are depicted in Table 1.

**Table 1:** Experimental conditions of bed bugs at different temperature and relative humidity.

Groups	Temperature (℃)	Relative Humidity (%)
Group-1	20	50
Group-2		70
Group-3		90
Group-4	25	50
Group-5		70
Group-6		90
Group-7	30	50
Group-8		70
Group-9		90

### Adult mortality and weight loss

Adult bed bugs (12–15 days old) (n = 20, Male/Female ratio 1:1) were given a rabbit blood meal (15 min) prior to initiation of the experiment. The weight of all the adults was taken before exposing them in temperature-humidity cabinet. For every group, three replicates were taken and checked every day to note down mortality. During the experimental period (4 weeks for adults) blood meal was given to them once in a week and total weight of the bugs for each replicate were taken before and after every blood meal. The average weight of bed bugs was taken every week and the weight loss was calculated by the formula mentioned in Eq. (1) below.

1 $$ {\text{Weight loss}} = {\text{W}}1 - {\text{W}}2{ }\left( {{\text{mg}}} \right) $$

where W1 = average weight of blood-fed bed bugs of day 0, W2 = average weight of unfed bed bugs of day 7.

### Egg laying and hatching

Female bed bugs start laying eggs after a few days of blood meal, on the paper harbourage that was replaced by the same type of paper on a daily basis and counted till 4 weeks. The data were represented as the average number of eggs laid for individual group. The papers were then kept in a disposable polypropylene petri-dish (8.5 cm diameter) with two small holes on the lid, under the same temperature and humidity condition. It was then observed on a daily basis for hatching initiation and completion. The percentage of hatching was calculated and data were represented as mean ± S.E.M. (standard error of the mean).

### Nymph mortality and molting

The nymphs (n = 30) were collected from the newly hatched eggs on the first week and kept in three different petri-dishes (10 nymphs per petri-dish). Nymphs were fed with blood meal (15 min) once in every 12 days and observed for molting and mortality. The molting of nymphs to their next stage was recorded when there was a first appearance of exoskeleton and noted as molting initiation, whereas when all the nymphs completed their molting was noted as the molting completion. The average days of molting initiation and completion were calculated by taking the mean values of initiation and completion for all stages.

### Statistical analysis

For adult mortality the comparisons were made between each group for male and female separately. For adult weight loss, percentage egg hatching, egg hatching initiation, egg hatching completion, percentage nymph mortality, nymph molting initiation, and nymph molting completion, the comparisons were made between each group. For egg laying, comparison was made only within a week between all the groups. All the data were represented as mean ± S.E.M. (standard error of the mean). The individual group’s significance for each individual parameter was analysed by one-way ANOVA followed by Tukey’s multiple comparison test where *p* < 0.05 has been considered as statistically significant.

## Results

### Adult mortality and weight loss

The adult mortality was observed on a daily basis for each replicate in every group and percentage mortality was calculated. The results were represented in Table 2 and were found statistically significant, for female (*p* < 0.001) and male bugs (*p* < 0.001). The lowest adult mortality of 13.33 ± 3.33% and 6.67 ± 3.33% was found at 25 °C with 90% RH after 4 weeks for female and male bed bugs, respectively. Mortality of 100% female bugs had occurred at 20 °C with 50% RH and 30 °C with 50% RH at the end of third week, while in male bed bugs it was observed at fourth week only for group 7. The weight loss of adult bed bugs was calculated from the weight of bed bugs before and after each blood meal for every week (Eq. 1). The loss of body weight was found statistically significant (*p* < 0.001).

**Table 2:** Percentage mortality and weight loss of adult bed bugs, values represented as mean ± S.E.M. (standard error of the mean).

Groups	Adult mortality percentage (%)		Adult weight loss (mg)
	Female	Male	
Group-1	100.00 ± 0.00^A^	93.33 ± 3.33^A^	5.88 ± 0.14
Group-2	53.33 ± 6.67^B, A^***	36.67 ± 3.33^B, A^***	5.63 ± 0.12
Group-3	26.67 ± 3.33^A^***^, B^**	16.67 ± 3.33^A^***^, B^*	4.96 ± 0.15
Group-4	86.67 ± 3.33^C^	60.00 ± 5.77^C, A^***	5.95 ± 0.19^A^
Group-5	36.67 ± 3.33^D, C^***	26.67 ± 3.33^D, C^***	5.71 ± 0.30
Group-6	13.33 ± 3.33^E, C^***^, D^**	6.67 ± 3.33^C^***^, D^*	5.05 ± 0.20^A^*
Group-7	100.00 ± 0.00^F^	100.00 ± 0.00^E, C^***	6.53 ± 0.24^B^
Group-8	73.33 ± 3.33^G, B^*^, D^***^, F^**	56.67 ± 3.33^F, B^*^, D^***^, E^***	5.87 ± 0.07
Group-9	43.33 ± 3.33^E^***^, F^***^, G^***	23.33 ± 3.33^E^***^, F^***	5.26 ± 0.15^B^**

The letters A, B, C, D, E, F, and G represents individual groups and letter with superscript *p < 0.05, **p < 0.01, and ***p < 0.001 represents the level of significance derived from one-way ANOVA followed by Tukey’s multiple comparison test.

### Egg laying

The average number of eggs laid by the female bed bugs in each exposure group were represented in Table 3. The highest numbers of eggs were laid by the bugs exposed to 25 °C with 90% RH on first and third week, i.e., 40.33 ± 1.86 and 39.67 ± 3.84. At 20 °C and 30 °C with 50% RH, eggs were collected up to third week as no female bed bugs survived beyond third week. The lowest numbers of eggs were collected at 20 °C with 50% RH. A significant increase in the average number of eggs on week 1 (*p* < 0.05) and week 2 (*p* < 0.001) was observed in the bugs exposed to 30 °C with 50% RH when compared to 20 °C with 50% RH.

**Table 3:** The average number of eggs laid in different groups were represented as mean ± S.E.M. (standard error of the mean) from week-1 to week-4.

Groups	Average number of eggs laid			
	Week-1	Week-2	Week-3	Week-4
Group-1	16.00 ± 1.53^A^	7.67 ± 0.33^A^	3.00 ± 0.58^A^	NA
Group-2	31.67 ± 2.19^A^	19.67 ± 2.19^B, A^*	8.33 ± 1.20^B^	3.67 ± 0.67^A^
Group-3	31.00 ± 1.53^A^**	15.67 ± 2.03^C^	19.00 ± 3.06^C, A^**^, B^*	19.67 ± 1.67^B, A^***
Group-4	27.67 ± 1.76^B, A^*	19.00 ± 1.00^D, A^*	8.33 ± 0.33^D^	3.00 ± 0.58^C^
Group-5	34.67 ± 2.40	33.33 ± 1.20^B^**^, D^**	21.33 ± 1.86^E, B^**^, D^**	11.33 ± 1.20^D, A^**^, C^**
Group-6	40.33 ± 1.86^B^*	35.00 ± 2.52^C^***^, D^***	39.67 ± 3.84^F, C^***^, D^***^, E^***	23.33 ± 2.33^C^***^, D^***
Group-7	28.67 ± 3.71^A^*	23.00 ± 2.65^A^***	3.67 ± 1.76^G^	NA
Group-8	30.33 ± 0.67	25.67 ± 2.60	21.67 ± 1.76^B^**^, G^***	9.00 ± 1.00
Group-9	36.67 ± 3.28	28.00 ± 2.00^C^**	19.00 ± 2.00^F^***^, G^**	13.67 ± 0.88^B^*

The letters A, B, C, D, E, F, and G represents individual groups and letter with superscript (*p < 0.05, **p < 0.01, and ***p < 0.001) represents the level of significance derived from one-way ANOVA followed by Tukey’s multiple comparison test. NA represents no egg available.

### Egg hatching

**Figure 2:**
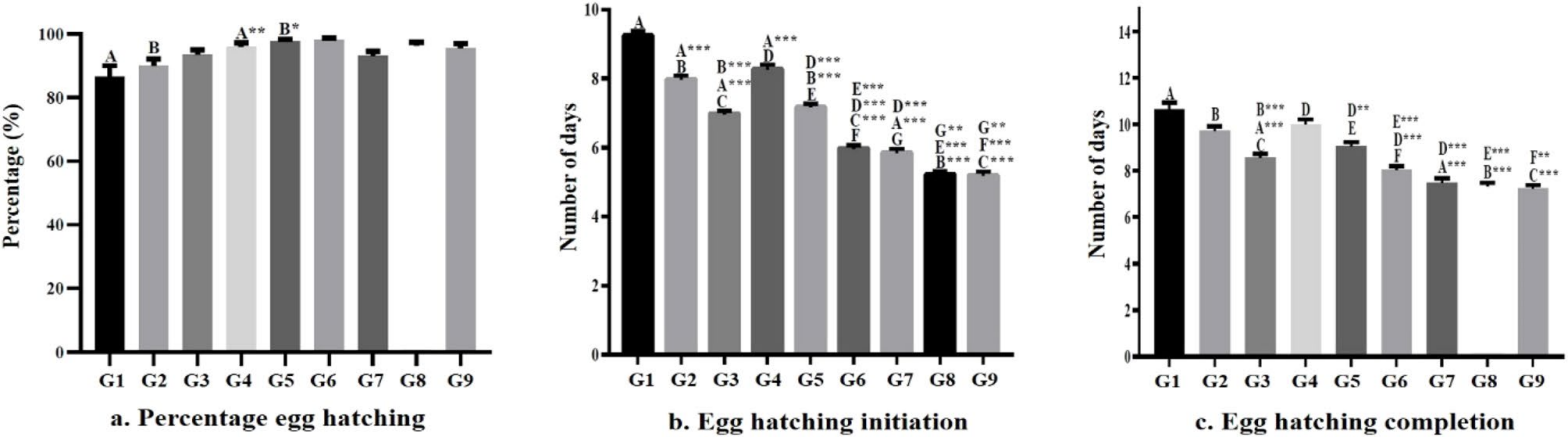
Graphical representation of the effect of temperature and relative humidity on percentage egg hatching (**a**), egg hatching initiation (**b**), and egg hatching completion (**c**). Each value was represented as mean ± S.E.M. (standard error of the mean). The letters A, B, C, D, E, F, and G represents the individual groups and the letters with superscript (*p < 0.05, **p < 0.01, and ***p < 0.001) represents the level of significance derived from one-way ANOVA followed by Tukey’s multiple comparison test.

The percentage egg hatching, average initiation and completion of hatching were represented in Fig. 2. The percentage hatching was found maximum at 25 °C with 90% RH i.e., 98.23 ± 0.58%. The egg hatching at 20 °C with 50% RH took maximum time (9.24 ± 0.14 days for initiation and 10.66 ± 0.28 days for completion) and the percentage hatching was also found lowest (86.58 ± 3.4%). Hatching of eggs at 30 °C with 90% RH was faster (5.19 ± 0.12 days for initiation and 7.23 ± 0.16 days for completion) in comparison with other groups.

### Nymph mortality

The mortality of nymphs was also observed daily for each group until they grown up to adults. The percentage mortality of nymphs was found to be similar for 20 °C with 50% RH, 25 °C with 50% RH group, and 30 °C with 90% RH i.e., 26.67 ± 3.33%. No significant changes in mortality were observed when humidity increases from 70 to 90% at 20 °C i.e., 23.33 ± 5.77%. But, when the temperature was increased from 20 to 25 °C for 70% RH, the percentage mortality was slightly decreased and the values were non-significant (*p* > 0.05) and reported to be 20.00 ± 5.77%. The group, 25 °C with 90% RH, highest number of nymphs survived till their adult stage and hence the percentage mortality was found to be minimum (13.33 ± 3.33%). The maximum nymph mortality was observed at 30 °C with 50% RH i.e., 36.67 ± 5.33%. When RH increased to 70% at 30 °C, percentage mortality decreased non-significantly (*p* > 0.05) and was found to be 33.33 ± 3.33.

### Nymph molting

**Figure 3:**
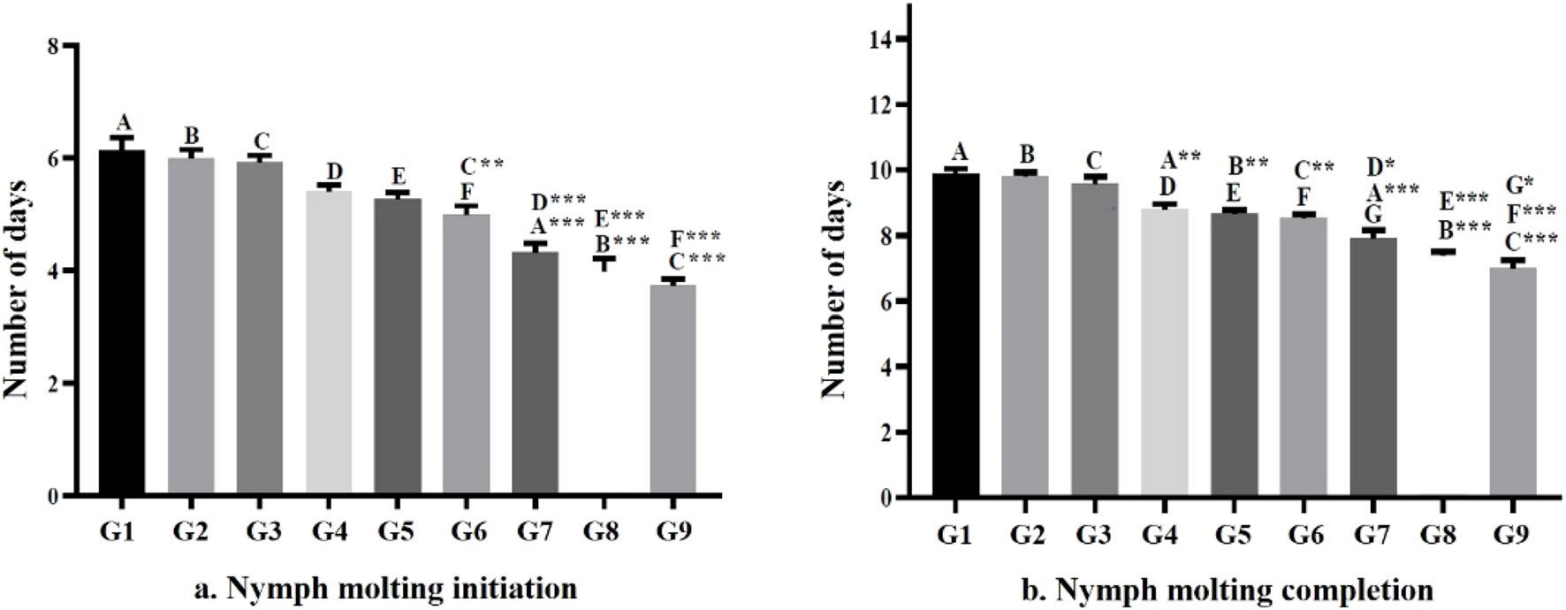
Effect of temperature and relative humidity on nymph molting. (**a**) represents the average number of days required for initiation of molting for all stages (first instar to fifth instar) after a blood meal, and (**b**) represents the average number of days to complete molting after a blood meal. Data are represented as mean ± S.E.M. (standard error of the mean). The letters A, B, C, D, E, F, and G represents the individual groups and the letters with superscript (*p < 0.05, **p < 0.01, and ***p < 0.001) represents the level of significance derived from one-way ANOVA followed by Tukey’s multiple comparison test.

The nymph molting was observed for its initiation and completion after every blood meal in each instar stage. The data for each group is represented in Fig. 3. The results were found statistically significant for molting initiation (*p* < 0.001) and for molting completion (*p* < 0.001). The molting initiation and completion time were minimum at 30 °C with 90% RH i.e., 3.73 ± 0.12 days and 7.00 ± 0.24 days respectively. In case of 20 °C with 50% RH, the molting initiation and completion time was found to be maximum i.e., 6.13 ± 0.23 days and 9.87 ± 0.17 days respectively.

## Discussion

The change in behavioural response with respect to variable temperature and relative humidity conditions are the major criteria that effect the bed bug population worldwide. They are known to cause severe mental trauma as well as physical discomfort to mankinds^13–16^. These types of challenges have aroused various questions in the scientific community regarding bed bug management. To eradicate the infestation, knowledge of its physiology, anatomy, disease vectoring conditions, and its controlling strategies are required to be understood at the earliest for which a good laboratory conditions is a pre-requisite. This study will put insight into unclenched temperature and RH conditions that will provide best culture acclimatization for *C. lectularius* L. species of bed bugs.

From the current study, it was observed that maximum mortality of adult bed bugs occurred in group 1 and group 7. As the humidity increases from 50 to 90%, the mortality rate has decreased significantly for both male and female bed bugs (*p* < 0.001) which is comparable with the previous findings^25–27^. Moreover, as the reproductive process reduces the agility and overall strength of female bed bugs, hence, they were more susceptible than the males as the survival rate of female bed bugs were noted to be less in every individual group.

Generally, after a blood meal, their weight increases almost five times and previous findings also revealed that due to dehydration and heat stress the body weight reduces eventually when exposed to higher temperature conditions^13,27–30^. In the current study, similar patterns of weight loss have been observed and found to be increasing with an increase in temperature and decreasing with an increase in RH as represented in Table 2. However, in Tukey’s multiple comparison test the weight loss for group 1, group 2, and group 3 was found to be non-significant (*p* > 0.05). This phenomenon can be explained due to the fact that at low temperature, the activity of bed bug is usually low which can cause conservation of body fluid^29^. Furthermore, a mortality of 100% for both females and males were seen for group 7, which is due to an increase in temperature and decrease in RH. Even the female bed bugs of group 1 and group 7 couldn’t survive beyond 3 weeks which can be ascertained due to its low humidity conditions. Thus, it clearly indicates that dehydration followed by weight loss plays an important role in the survival of adult bed bugs.

From the previous studies, it was evident that at 23 °C with 90% RH, female *C. lectularius* L. can lay 5–8 eggs per week, which can reach up to 100–150 in their entire life time^31–33^. The newly emerged nymphs generally take a blood meal and molts their keratinous outer shell so as to reach their next stage of development and continue the nymphal maturation stage, finally growing into an adult^2^. Herein, the egg laying was found to be maximum for group 6 and can be considered to be the most favourable environment for egg laying. Nymphs emerge from fertilized eggs within 5–12 days depending upon the environmental conditions^34,35^. Thus, hatching initiation was observed to understand the time required for embryonic development under different conditions. The percentage egg hatching, on the other hand has been found to be maximum in group 6. Therefore, it can be considered that group 6 (25 °C with 90% RH) is probably the ideal environment for egg incubation too. The nymph mortality has been observed all through their progressive development and the highest mortality was found at group 7 which was quite similar with the observed adult mortality and adult weight loss patterns. With the increase in relative humidity, the nymph mortality had slightly decreased. However, it was seen that with an increase in temperature, the mortality percentage remains almost the same. This suggests that relative humidity has more profound impact on the survival rate of nymphs than the temperature variations.

**Figure 4:**
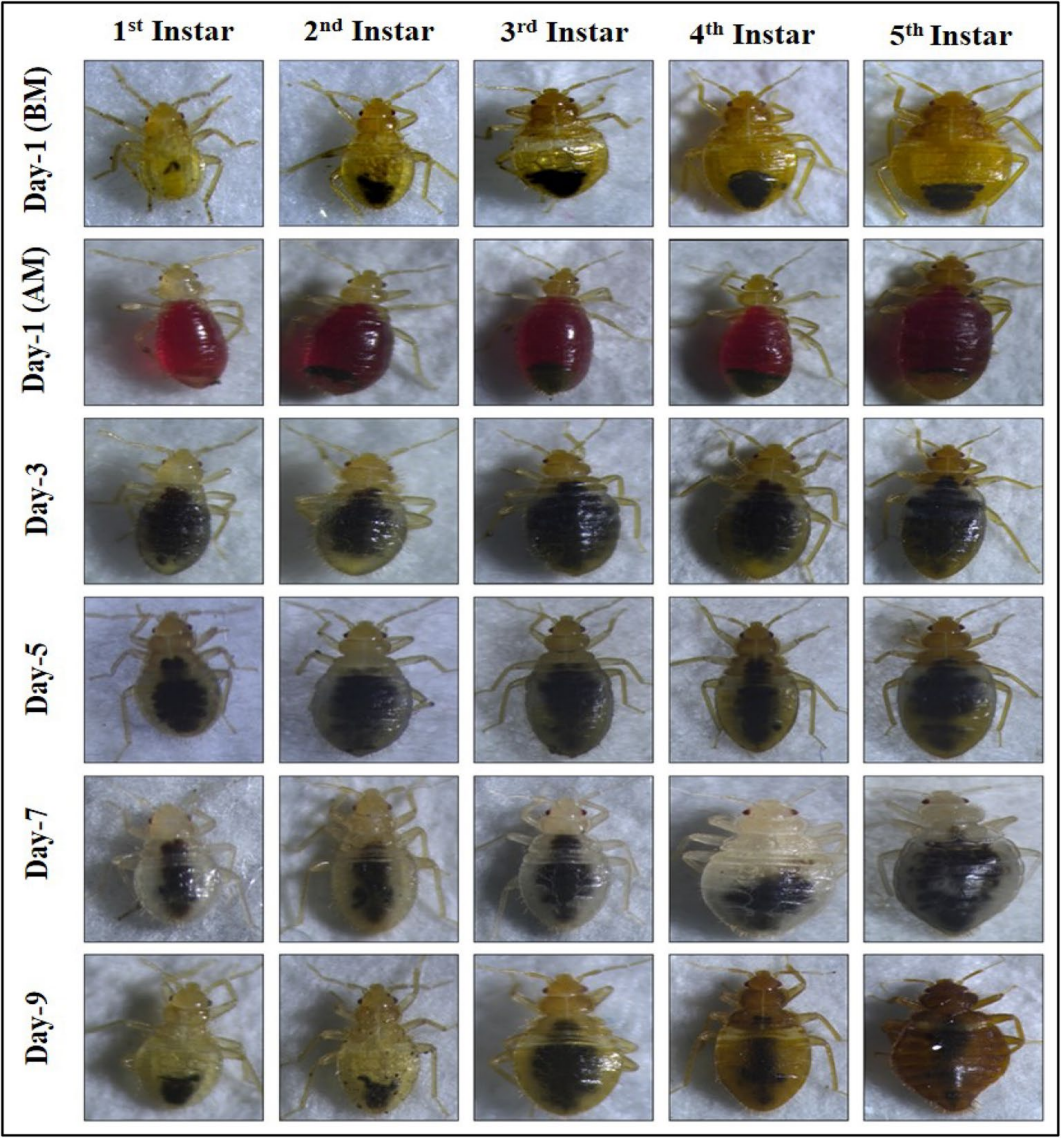
Pictorial representation of different stages of development of *C. lectularius* L. The columns first to fifth represents the nymphal stages (first instar to fifth instar), and the rows represents the day wise changes at every stage. Row 1 and 2 depict the nymphs of all stages before blood meal (BM) and after blood meal (AM), while row 3 and 4 represent the changes taking place after blood meal at day-3 and day-5 respectively. A clear visualisation of changing of old keratinous structure to new apparently whitish skin structure indicates the completion of molting and transition to their next stage.

One of the vital physiological transitions that occur in nymphs is molting^13^. They usually change their exoskeleton after every 3–7 days following a blood meal which takes almost 40–60 days to become an adult^2,3,34^. In this particular study, molting pattern (both initiation and completion) were observed in terms of days. The data were analysed and found significant in most of the cases when the temperature and humidity conditions were compared, and reported in Fig. 3. The pictorial representation that the bed bug nymphs go through during their development has been shown in Fig. 4. It has been observed that with an increase in temperature from 20 to 30 °C and RH from 50 to 90%, both molting initiation and molting completion significantly decreased (*p* < 0.001). Thus, it can be said that temperature and RH have profound role in the stages of nymphal developments.

## Conclusion

The effect of temperature and relative humidity was observed throughout the life cycle of *C. lectularius* L. After the completion of this study, it can be stated that at 25 °C with 90% RH, maximum number of adults and nymphs survived during entire study period, also egg laying and percentage egg hatching was maximum in this condition. But in case of initiation and completion of both egg hatching and nymph molting the most favourable condition was 30 °C with 90% RH, but the mortality of adults was found almost 4 times higher than 25 °C with 90% RH condition. Thus, it can be concluded that 25 °C with 90% RH will be the most suitable condition for rearing of adult bed bugs, and 30 °C with 90% RH will be the most favourable condition for rapid growth of nymphs, provided the frequency of blood meal should be increased to obtain better results.

## Data Availability

The datasets used and/ or analyzed during the current study are available from the corresponding author on reasonable request.
